# A subunit of the oligosaccharyltransferase complex is required for interspecific gametophyte recognition in *Arabidopsis*

**DOI:** 10.1038/ncomms10826

**Published:** 2016-03-11

**Authors:** Lena M. Müller, Heike Lindner, Nuno D. Pires, Valeria Gagliardini, Ueli Grossniklaus

**Affiliations:** 1 Department of Plant and Microbial Biology and Zürich-Basel Plant Science Center, University of Zürich, Zollikerstrasse 107, 8008 Zürich, Switzerland

## Abstract

Species-specific gamete recognition is a key premise to ensure reproductive success and the maintenance of species boundaries. During plant pollen tube (PT) reception, gametophyte interactions likely allow the species-specific recognition of signals from the PT (male gametophyte) by the embryo sac (female gametophyte), resulting in PT rupture, sperm release, and double fertilization. This process is impaired in interspecific crosses between *Arabidopsis thaliana* and related species, leading to PT overgrowth and a failure to deliver the sperm cells. Here we show that *ARTUMES* (*ARU*) specifically regulates the recognition of interspecific PTs in *A. thaliana*. *ARU*, identified in a genome-wide association study (GWAS), exclusively influences interspecific—but not intraspecific—gametophyte interactions. *ARU* encodes the OST3/6 subunit of the oligosaccharyltransferase complex conferring protein *N*-glycosylation. Our results suggest that glycosylation patterns of cell surface proteins may represent an important mechanism of gametophyte recognition and thus speciation.

## Abstract (web summary)

Species-specific gamete recognition is needed to maintain species boundaries. Here, Müller *et al*. show that *ARTUMES* regulates pollen tube recognition between different *Arabidopsis* species, representing the first gene known to exclusively influence inter- but not intraspecific gamete interaction in plants.

## Graphical abstract

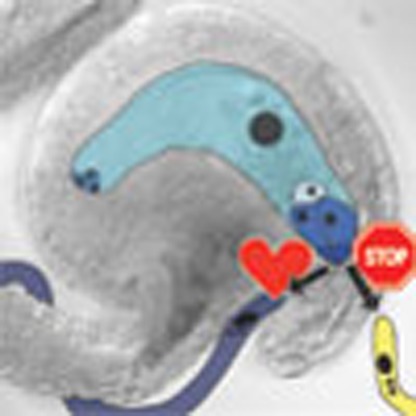

Species evolve and are maintained by a variety of hybridization barriers that prevent interspecific gene flow and thus the formation of potentially unviable or sterile hybrids^1^. To date, the molecular basis of hybridization barriers is still poorly understood. In plants, such barriers can either act before (pre-pollination barriers) or after pollination (post-pollination barriers). Pre-pollination barriers can be spatial or temporal patterns preventing plants from being pollinated by pollen from a different species, whereas post-pollination barriers come into play only after an interspecific pollination event occurs and can be further divided into pre- and post-zygotic barriers^2^. The latter usually act at the genomic level (for example, incompatibilities leading to hybrid lethality or sterility), while pre-zygotic barriers prevent the formation of a zygote and usually rely on direct cell–cell communication between the male and female tissues. Most species pairs are isolated by a complex interplay of different types of isolation barriers. Whereas barriers that prevent fertilization (both pre-pollination and pre-zygotic barriers) often represent the most important means to reduce interspecific gene flow, post-zygotic hybridization barriers appear to contribute less to reproductive isolation in many species pairs^3^.

In plants, successful fertilization starts with the deposition of intraspecific (same-species) pollen (male gametophyte) onto the stigma of a gynoecium. The subsequent steps involve extensive communication between the male and female tissues, leading to pollen adherence, hydration, and the germination of a pollen tube (PT). Within its cytoplasm, the tip-growing PT transports the two sperm cells through the transmitting tract of the pistil to the embryo sac (female gametophyte), which is deeply embedded in the ovule, the precursor of seed. During its journey, the PT is guided towards the embryo sacs by attractants secreted by female tissues^4^. On arrival at the embryo sac, communication between the PT and the synergid cells of the female gametophyte is initiated (Fig. 1a). The two synergid cells are located at the micropylar end of the embryo sac and possess a secretory region characterized by membrane invaginations and thickened cell wall structures^5^. This so-called filiform apparatus is the first point of contact between the male and female gametophytes, which communicate in preparation for penetration of the receptive synergid cell by the PT, PT rupture, sperm release, and double fertilization^6^. While one sperm fuses with the egg cell to form the diploid zygote, the other fertilizes the homo-diploid central cell to produce the triploid endosperm, an embryo-nourishing tissue. The communication process between the male and female gametophytes leading to PT rupture and sperm cell discharge is known as PT reception, and its success or failure is under female gametophytic control^6^.

However, if a pollen grain originating from a different species (interspecific pollination) is placed on a plant's stigma, all the communication processes described above have the potential to act as pre-zygotic post-pollination barriers. Several studies describe a species-preferential behaviour of molecular factors involved in pollen adherence to the stigma, PT growth, and PT guidance towards the ovules^4^,^7^,^8^,^9^,^10^,^11^. In interspecific crosses between closely related Ericaceae or Brassicaceae, respectively, hybridization barriers act at the stage of PT reception^12^,^13^. In such crosses, PTs are properly targeted to the female gametophyte but, upon arrival at the embryo sacs, interspecific PTs are not recognized and fail to arrest growth and discharge their sperm. Instead, they continue growing inside the embryo sacs (referred to as PT overgrowth) and cannot effect double fertilization. Therefore, we consider PT reception to be an integral part of the hybridization barrier in these species. Interspecific PT overgrowth phenocopies the female gametophytic mutants *feronia/sirène* (*fer/srn*), *lorelei* (*lre*), *nortia* (*nta*), *turan* (*tun*), *evan* (*evn*), and *Zea mays embryo sac 4* (*ZmES4*) RNAi-lines^13^,^14^,^15^,^16^,^17^,^18^,^19^,^20^, which are defective in the reception of intraspecific PTs. In addition, *FER* has been proposed to be involved in interspecific PT recognition^13^, and there is evidence that *ZmES4* is sufficient to trigger PT growth arrest and rupture in a species-preferential manner^20^.

Despite the rapid advance in our understanding of the molecular basis of intraspecific PT reception^21^, the genetic basis of post-pollination hybridization barriers remains largely unknown. All molecular factors that have so far been described to be involved in pre-zygotic species-discrimination, including species-preferential pollen adherence, PT guidance, growth, and reception, act primarily during intraspecific pollination and have additional species-preferential effects^4^,^7^,^8^,^9^,^10^,^11^. Here, we report the identification of the first gene required exclusively for inter- but not for intraspecific pollination, thus likely representing a specific component for the establishment of a hybridization barrier. By making use of the striking natural variation of *A. thaliana* accessions in interspecific PT reception, we identified *ARTUMES* (*ARU*) as an indispensable factor for the recognition of interspecific *A. lyrata* PTs by *A. thaliana* embryo sacs. In contrast, *aru* mutants do not affect gametophytic communication in intraspecific *A. thaliana* crosses. *ARU* encodes the OST3/6 subunit of the oligosaccharyltransferase complex, which is known to regulate site- and substrate-specific *N*-glycosylation of proteins in yeast^22^,^23^ and a similar substrate specificity has been reported for *A. thaliana* OST3/6 (ref. 24). Thus, a possible mechanism for the discrimination of inter- and intraspecific PTs may depend on the species-specific glycosylation of proteins on the surface of the synergid cells of the female gametophyte.

## Results

### PT overgrowth restricts gene flow between *Arabidopsis* species

**Figure 1:**
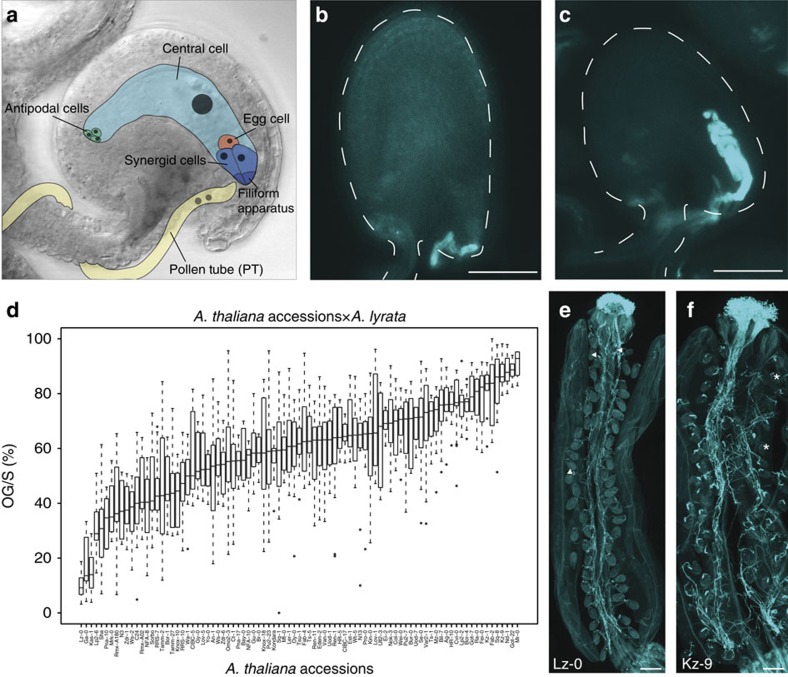
PT reception in interspecific crosses. (**a**) Diagram of the female gametophyte with its cell types. The synergids with their membrane-rich filiform apparatus are crucial for communication with the arriving PT. (**b**) Ovule with normal PT reception, visualized by callose staining of the PT cell walls with Aniline Blue. The PT stopped its growth and ruptured. Dashed line indicates outline of the ovule. (**c**) Ovule with PT overgrowth. The PT continues growing inside the female gametophyte. Dashed line indicates outline of the ovule. (**d**) Natural variation in the proportion of ovules with PT overgrowth per silique (OG/S) in 86 *A. thaliana* accessions that were pollinated with *A. lyrata* pollen. OG/S varies between 10% and more than 90%, depending on the genotype of the mother. (**e**) A silique of Lz-0 pollinated with *A. lyrata* pollen. Most of the ovules show normal PT reception. Ovules with PT overgrowth are marked with an arrowhead. (**f**) A silique of Kz-9 pollinated with *A. lyrata* pollen. Most of the ovules display PT overgrowth. Asterisks mark ovules with normal PT reception. Per accession and per pollen donor, 5–10 siliques were analysed. Scale bars, 50 μm (**b** and **c**), 250 μm (**e** and **f**).

Self-fertilizing *A. thaliana* and its outcrossing relative *A. lyrata* are separated by strong pre-pollination barriers due to their different mating systems^1^. In addition, they are isolated by post-pollination barriers based on direct male–female interactions. Although *A. thaliana* (Col-0) pollen germination is inhibited at the *A. lyrata* stigma, *A. lyrata* PTs are guided towards *A. thaliana* embryo sacs, but PT reception fails (Fig. 1c, as opposed to Fig. 1b showing successful PT reception, Supplementary Fig. 1). Such unilateral incompatibility is similar to that observed in other crosses between self-compatible and self-incompatible species^25^. We also observed PT overgrowth in interspecific crosses between *A. arenosa* and *A. lyrata* (Supplementary Fig. 2), between which natural gene flow occurs^26^. This finding indicates that *A. lyrata* PT overgrowth in *A. thaliana* ovules does not only occur between species that do not interbreed in nature (*A. thaliana* × *A. lyrata*) but also between species that are only partially reproductively isolated and do interbreed (*A. arenosa* × *A. lyrata*).

### Natural variation in interspecific PT reception

To analyse interspecific hybridization barriers within the genus *Arabidopsis*, we assessed PT overgrowth in 86 *A. thaliana* accessions that were pollinated with *A. lyrata* pollen (Supplementary Table 1). PTs were visualized by staining callose in PT cell walls with aniline blue. We scored the proportion of ovules that failed to recognize interspecific PTs—leading to PT overgrowth—in relation to the total number of ovules that attracted a PT in a silique (overgrowth per silique, OG/S). We found a striking variation in the ability to recognize interspecific PTs between different *A. thaliana* accessions, with OG/S ranging from about 10 to 90% (Fig. 1d, broad-sense heritability *H*^2^=0.7). Examples of accessions with extreme phenotypes are Lz-0 (10% OG/S, *n*=12 siliques) and Kz-9 (87.3% OG/S, *n*=10; Fig. 1e,f). There is no obvious correlation between the geographical origin of the accessions and their phenotype (Supplementary Fig. 3).

**Figure 2:**
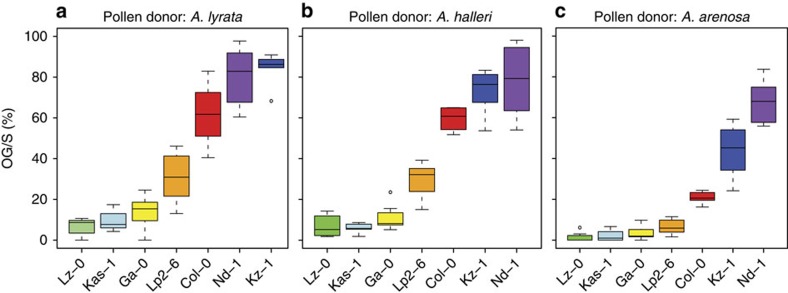
Natural variation in PT reception with different interspecific pollen donors. (**a**) A subset of *A. thaliana* accessions pollinated with *A. lyrata* pollen. Per accession, four to eight siliques were analysed. Box plots are ordered by the mean OG/S value and colour-coded to facilitate comparison with **b** and **c**. (**b**,**c**) The same subset pollinated with pollen from *A. halleri* (**b**) and *A. arenosa* (**c**). The accessions show comparable OG/S with all three interspecific pollen donors.

To analyse whether the variation in the ability to recognize interspecific PTs is species-dependent, we pollinated a subset of *A. thaliana* accessions with low or high OG/S in crosses with *A. lyrata* (Lz-0, Kas-1, Ga-0, Lp2-6 and Col-0, Kz-1, Nd-1, respectively) also with pollen of *A. halleri* and *A. arenosa*. Although OG/S in the accessions pollinated with *A. lyrata* or *A. halleri* pollen was highly comparable (Fig. 2a,b), the values were slightly lower for all accessions when pollinated by *A. arenosa* (Fig. 2c), indicating that *A. thaliana* recognizes *A. arenosa* PTs better than those of *A. lyrata* or *A. halleri*. However, accessions showing very low or high OG/S in crosses with *A. lyrata*, respectively, displayed a similar phenotype with *A. halleri* and *A. arenosa* as pollen donors, suggesting a common molecular PT reception mechanism for all three species. Thus, PT overgrowth is a hallmark of interspecific crosses with close Brassicaceae relatives and not a species-specific feature of *A. thaliana* and *A. lyrata*.

To investigate whether intraspecific PT reception was affected in accessions with high OG/S (Col-0, Kz-1, Kz-9, Nd-1, Fei-0, and Sq-8), we crossed them with *A. thaliana* pollen (from both low and high OG/S accessions). Intraspecific PT reception was normal in all the tested accessions (Supplementary Fig. 3), indicating that high OG/S frequencies result from a failure in the recognition of interspecific PTs only, and are not due to a general defect in PT reception.

### *ARTUMES* regulates inter- but not intraspecific PT reception

**Figure 3:**
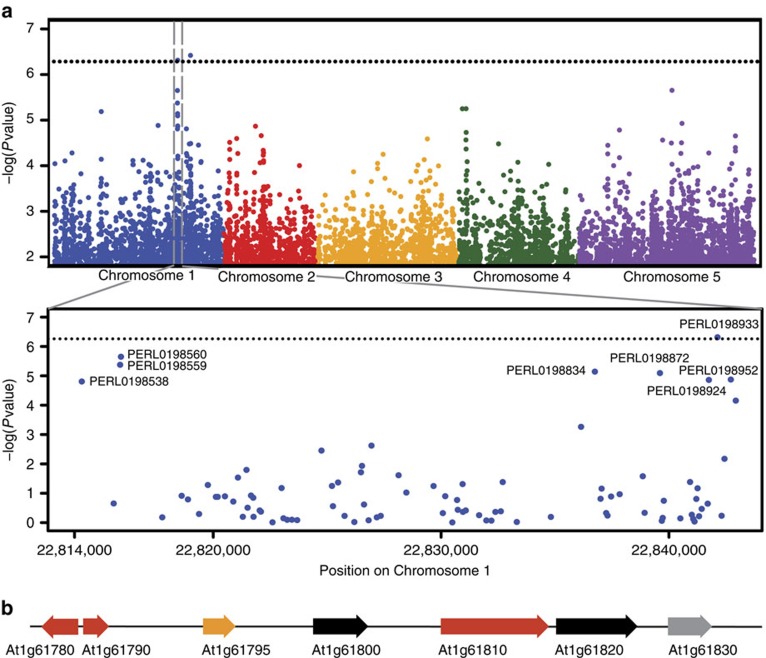
GWAS identifies an associated region on chromosome 1. (**a**) Manhattan plot showing a peak on chromosome 1 (grey box) with its highest correlated SNP showing significance at *P*<0.1 (after Bonferroni correction; dotted line). The peak corresponds to a 28 kb region spanning position 22,842,689–22,814,316 (magnified in the second panel). The eight SNPs that were identified to be among the 20 most highly correlated ones in the GWAS are annotated with their PERL identifiers. (**b**) Genes and pseudogenes (grey) in the 28 kb region. Genes expressed in synergids are marked in red, genes without available expression data are in orange^32^.

To identify loci causing the variation in interspecific PT reception in *A. thaliana*, we used publicly available single-nucleotide polymorphism (SNP) data from the 86 accessions to perform a genome-wide association study (GWAS)^27^. To date, most GWAS in *Arabidopsis* have identified previously known candidate genes, with only a few studies identifying novel regulatory genes in the respective pathways^28^,^29^. Applying the GLM function implemented in TASSEL^30^, we identified a region on chromosome 1 containing 8 of the top 20 SNPs with the highest correlation to the OG/S trait (Fig. 3a and Supplementary Table 2). This 28-kb region (positions 22,814,316 to 22,842,689) contains six genes and one pseudogene (Fig. 3b). Interestingly, calculation with mixed linear models that simultaneously correct for population structure and unequal genetic relatedness between individuals masked the peak, whereas it could be detected—although below the significance threshold—using a step-wise multi-locus mixed model (MLMM) specifically designed for mapping complex traits^31^ (Supplementary Fig. 4). With each step of MLMM, new peaks appear, consistent with a multigenic basis for interspecific PT reception.

**Figure 4:**
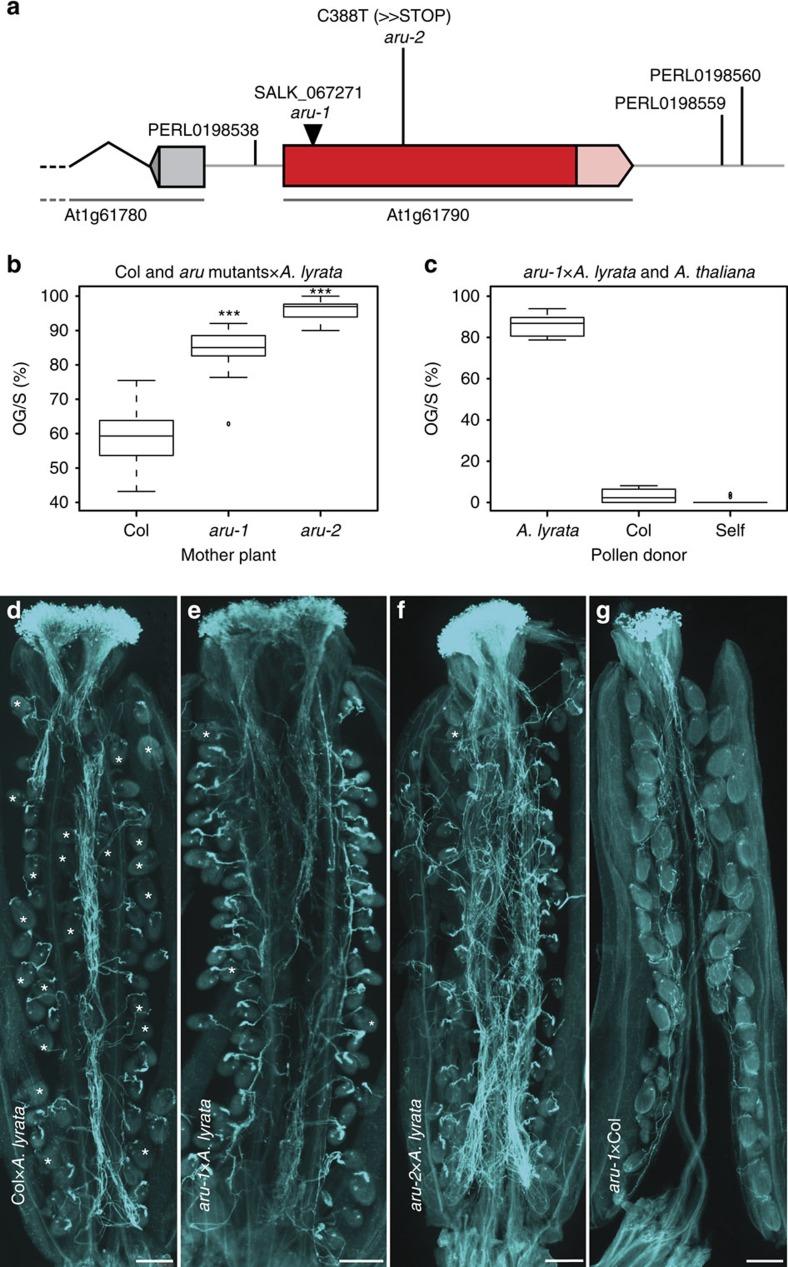
*ARTUMES* mutants are impaired in interspecific PT reception. (**a**) Genomic region of *ARTUMES (At1g61790)* with the two mutant alleles *aru-1* and *aru-2*, and the surrounding polymorphisms identified by GWAS. (**b**) PT overgrowth of Col-0 wild-type (*n*=28 siliques) and *aru* mutant plants in interspecific crosses. Both *aru* mutant alleles show significantly higher proportions of ovules with PT overgrowth per silique (OG/S, Student's *t*-test ***P<0.001; *n*=18 and 20 siliques, respectively). (**c**) PT overgrowth of *aru-1* in inter- and intraspecific crosses. The mutant is impaired in interspecific crosses with *A. lyrata* pollen (*n*=9 siliques), but not in intraspecific crosses with Col-0 (*n*=6) or self pollen (*n*=9). (**d**) A silique of Col-0 pollinated with *A. lyrata* pollen. Ovules with normal PT reception (marked with asterisks) and with PT overgrowth are visible. (**e**,**f**) *aru-1* and *aru-2* siliques pollinated with *A. lyrata* pollen. Both mutant alleles show high proportions of ovules with PT overgrowth in interspecific crosses. Ovules with normal PT reception are marked with asterisks. (**g**) A silique of *aru-1* pollinated with intraspecific Col-0 pollen. All ovules display normal PT reception. Scale bars, 250 μm.

To narrow down the 28-kb candidate region to a single gene, we analysed OG/S in T-DNA insertion lines of three synergid-expressed genes^32^ in this region because the synergids control PT reception (*At1g61780, At1g61790* and *At1g61810*; as well as *At1g61795*, for which no expression data was available; Supplementary Fig. 5). In a homozygous T-DNA insertion allele disrupting the coding sequence of *At1g61790* (Fig. 4a), an average of 84.3% (*n*=18 siliques) of ovules display *A. lyrata* PT overgrowth, significantly more than in the Col-0 wild-type control (58.7% OG/S, *n*=28, Student's *t*-test *P*<0.001, Fig. 4b,d,e). We named the *At1g61790* gene, which has previously been described as *OST3/6* based on homology^24^, *ARTUMES* (*ARU)* after the Etruscan goddess of night, nature, and fertility^33^. The T-DNA allele was denoted *aru-1.* A second mutant allele, *aru-2*, carrying an EMS-induced premature stop-codon after aa residue 129 (ref. 34), also showed an increase in interspecific OG/S (96.1%, *n*=20, Fig. 4a,b,f). Likewise, *aru-1* mutants pollinated with *A. arenosa* pollen showed significantly more ovules with PT overgrowth (67.6% OG/S, *n*=8) than the wild type (34.9% OG/S, *n*=12, Student's *t*-test *P*<0.001, Supplementary Fig. 6), suggesting a common basis for interspecific PT recognition. In contrast, *aru* mutant ovules have no problem recognizing and receiving intraspecific PTs from *A. thaliana* (Fig. 4c,g), indicating that the PT reception pathway is fully functional within the species.

**Figure 5:**
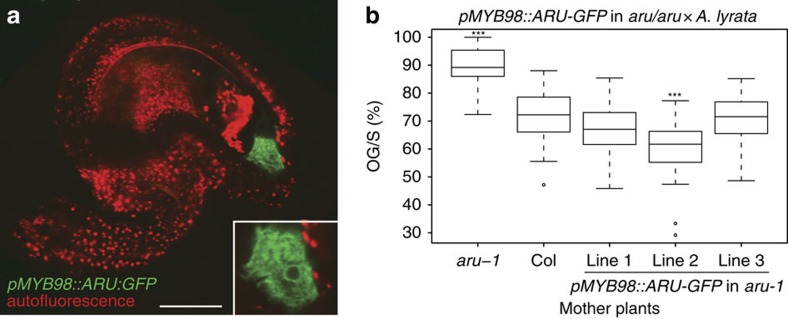
Synergid-specific expression of *ARTUMES* complements the mutant phenotype. (**a**) An ovule expressing *pMYB98::ARU-GFP* in the synergids. Inset: *ARU-GFP* localizes to perinuclear structures resembling the ER. (**b**) PT overgrowth in interspecific crosses using *aru-1* (*n*=22 siliques), Col-0 (*n*=28), and three independent transformant lines of *pMYB98::ARU-GFP* in the *aru-1* background as mother plants and *A. lyrata* as pollen donor. All the three transformant lines complement the mutant phenotype (*n*=16, 22, and 27 siliques, respectively); line 2 shows even lower OG/S than the wild type. Significance levels in comparison with Col-0 (Student's *t*-test ***P<0.001). Scale bar, 50 μm.

To confirm that *ARU* function is required for interspecific PT recognition in the synergids, we expressed an ARU-GFP translational fusion protein under the control of the *MYB98* and *FERONIA* promoters (*pMYB98::ARU-GFP* and *pFER::ARU-GFP*) in *aru-1* mutants. These promoters are highly active in synergids^13^,^35^, and in ovules the strongest ARU-GFP signal was detected in these cells (Fig. 5a and Supplementary Fig. 7). ARU-GFP localized to perinuclear structures resembling the ER in synergids (Fig. 5a, inset), and co-localized with an ER-marker in transiently transformed onion epidermal cells (Supplementary Fig. 7). These results are consistent with the previously reported ER-localization of ARU-GFP in infiltrated tobacco leaves^24^. Mutant *aru-1* plants expressing a functional copy of ARU-GFP in their synergids displayed wild-type-like PT reception in interspecific crosses (Fig. 5b and Supplementary Fig. 7), indicating that *ARU* expression in synergid cells is sufficient to complement the *aru* mutant phenotype. Consistent with this, the ARU-GFP translational fusion protein driven by the endogenous promoter (*pARU::ARU-GFP*) is highly expressed in wild-type synergids (Supplementary Fig. 7), suggesting an important role for *ARU* in these cells. Functional complementation of the *aru-1* mutant was also observed when *ARU* was driven by the endogenous promoter in a construct also containing 865 bp downstream sequence (*pARU::ARU*), although there was more line-to-line variability than when the *pMYB98* promoter was used (Supplementary Fig. 7). Since *ARU* is not fused to a fluorescent tag here, technical difficulties in measuring synergid specific expression of the transgene make it hard to explore this difference experimentally. However, it is conceivable that additional regulatory sequences, up- or downstream of the *ARU* coding sequence that are present in the *pARU::ARU* construct but not in *pMYB98::ARU-GFP* or *pFER::ARU-GFP*, contributed to the observed phenotypic variability.

### SNPs around *ARTUMES* correlate with phenotypic variation

We assessed the correlation of amino acid differences in the *ARU* coding sequence in all accessions to determine whether differences in the protein sequence could explain the phenotypic variation. Within this population, we detected a total of 10 amino acid differences, four of which are significantly correlated to variation in OG/S (Pearson's correlation coefficient *R*, *P*<0.05, Supplementary Data 1). However, differing residues had similar chemical properties, implying small, if any, differences in protein function. Alternatively, differential expression levels could cause the observed phenotypic variation. Therefore, we examined alignments of 1,000 bp up- and downstream sequence of *ARU*. We found 9 of 49 and 39 of 79 upstream and downstream SNPs, respectively, to be correlated with phenotypic variation (*P*<0.05, Supplementary Data 1), suggesting that phenotypic variation could be due to differences in gene expression. To investigate this further, we used RNA extracts from pistils and ovules collected 2 days after emasculation from selected accessions (Lz-0, Kas-1, Ga-0 and Col-0, Nd-1, Fei-0, Kz-1, Kz-9) for quantitative real-time PCR and digital droplet PCR, respectively^36^. We found *ARU* mRNA levels differed between accessions (Supplementary Fig. 8), but they did not correlate with the OG/S phenotype among the selected set of accessions. Because we used RNA from whole pistils and ovules, we cannot exclude the possibility that *ARU* is differentially expressed in synergids only, where it is required and sufficient for interspecific PT reception. In addition, post-transcriptional regulation of gene expression could play a role in mediating ARU protein levels. *ARU* has a 410 bp long 3′-UTR (3′-untranslated region)^37^ containing 17 SNPs that are correlated with the OG/S phenotype and might contribute to accession-specific differences in ARU protein levels.

To further investigate the role of *ARU* in different accessions, we transformed high OG/S accessions (Fei-0, Kz-1, Kz-9) with *pMYB98::ARU-GFP* to ensure strong expression in synergid cells and assessed interspecific PT reception in these lines. Of several independent transformants of all accessions, none showed a significant reduction of OG/S (Supplementary Fig. 8), which would be expected if low expression of *ARU* alone would be the cause of impaired interspecific PT reception in these accessions. Similarly, the *ARU* allele (including 1,492 bp up- and 865 bp downstream sequence) from Ga-0 could not better complement the *aru* mutant—which is in the Col-0 background—than the Col-0 allele, as it would be expected if differences in *ARU* alone caused the phenotypic difference between Ga-0 and Col-0 (Supplementary Fig. 7). Although these experiments could not establish a mechanistic link between variation in *ARU* and variation in OG/S, they are not inconsistent with the hypothesis that *ARU* is differentially regulated in *A. thaliana* accessions. Because *ARU* is likely only one of several factors involved in interspecific PT reception, we cannot rule out that crucial epistatic interactions with other factors were missing in the particular accessions we tested. At present, it is conceivable that population structure or co-segregating SNPs, which do not directly influence *ARU* expression or protein function, caused the GWAS peak on chromosome 1 and that *ARU* itself—although undoubtedly involved in interspecific PT reception—is not responsible for the observed natural variation among the 86 accessions tested. We consider co-segregating SNPs a highly unlikely explanation, however, because mutations disrupting other genes in the region identified by GWAS did not show a phenotype in interspecific crosses with *A. lyrata* pollen.

### Signatures of selection at the *ARTUMES* locus

Genes involved in reproductive isolation and speciation are often subject to selective pressures driving rapid divergence^38^. We tested *ARU* plus 1,000 bp up- and downstream sequence for signatures of positive selection by estimating Tajima's D^39^ for a set of 96 *A. thaliana* accessions^40^, including all accessions used in this study. A negative D is due to an excess of low frequency polymorphisms that can be caused by positive selection on the locus or by population expansion. Tajima's D was −2.07 for the 1,000 bp upstream of the translation start, −1.57 for the coding sequence and −1.58 for the 1,000 bp downstream of the *ARU* stop-codon. All values significantly deviate from the neutral model (*P*<0.05), but do not fall into the 5% tail of the estimated genomic distribution of D in *A. thaliana*^40^ (cut-off value: −2.08). Thus, although the 1,000 bp upstream region was very close to this cut-off, the observed negative values might be influenced by demographic factors that shaped the entire genome rather than selective pressure acting on the *ARU* locus. In addition, we estimated Fay and Wu's H, another test statistic to detect positive selection^41^, which is not as sensitive to demographic factors as Tajima's D^42^. All values for H were strongly negative (upstream region: −20.20, coding sequence: −21.79, downstream region: −20.73, *P*<0.02), and fall into the very extreme tails of both an empirical and a simulated distribution of H calculated with 12 accessions, which represent a world-wide distribution and are a subset of the accessions used in this study^42^. Such strongly negative values for H indicate that positive selection may indeed have acted on each of the regions of the *ARU* locus. The fact that D for the upstream region was very close to the 5% tail and the strongly negative values for H provide evidence for possible positive selection and indicate that *ARU* may have undergone a recent selective sweep. This is consistent with the lack of variation in *ARU* amino acid sequence and expression level but is difficult, although not impossible, to reconcile with the fact that we identified *ARU* in a GWAS for variation in the OG/S phenotype. As pointed out above, this may be due to epistatic interactions with additional factors involved in this complex process. Nevertheless, as in animal speciation genes, selective pressures appear to have contributed to shaping the genetic basis that underlies interspecific PT reception in *A. thaliana*.

### Interspecific PT reception depends on protein *N*-glycosylation

*ARU* encodes the OST3/6 subunit of the hetero-oligomeric plant oligosaccharyltransferase complex (OST), which catalyses the co- or posttranslational transfer of pre-assembled carbohydrate oligomers (Glc_3_Man_9_GlcNAc_2_) to asparagine (N) residues of polypeptides^43^. *N*-glycosylation affects the substrate protein's folding, targeting, and/or processing through the ER. Subsequently, the N-linked glycan can be modified in the Golgi apparatus in a cell-type and species-specific manner, accounting for the functionality and binding specificity of the glycoprotein^43^. The yeast OST consists of eight subunits and the homologues of OST3/6, Ost3p and Ost6p, differ in their protein substrate and site-specific glycosylation efficiency^22^,^23^.

In plants, OST3/6 confers similar substrate specificity since in the *A. thaliana ost3/6 (aru)* mutant only a subset of glycoproteins is misglycosylated and therefore non-functional^24^. Among these are the pathogen-associated molecular pattern (PAMP) receptor kinase EF-TU RECEPTOR (EFR), and KORRIGAN1, an endo-β-1,4-glucanase involved in cellulose biosynthesis. In line with this, *aru* has previously been identified in an EMS-screen for cell wall mutants^34^. Some of the known members in the PT reception pathway, *FER* and *NTA*, have been implicated in the perception of cell wall perturbations, pathogen resistance, and innate immunity^17^,^44^,^45^. After pollination with *A. lyrata*, *fer* heterozygous mutants show higher OG/S (74.9%) than wild-type segregants (61.2%, *P*<0.01, Supplementary Fig. 9), suggesting that inter- and intraspecific PT reception both involve the *FER* pathway. Moreover, FER (a receptor-like kinase with an extracellular malectin-binding domain) and LRE (a glycosylphosphatidylinositol-anchored protein) are likely to be glycosylated^13^,^16^,^46^ and could be substrates of ARU. To test this, we analysed the expression and localization of fluorescent FER and LRE fusion proteins in *aru* ovules. We included NTA, which itself does not contain any putative glycosylation sites but whose subcellular localization depends on *FER* signalling^17^. All fusion proteins displayed a wild-type-like subcellular localization in the synergids of *aru* embryo sacs: FER-GFP and LRE-Citrine were observed at the micropylar end of the synergids, and NTA-GFP was re-localized there upon PT arrival (Supplementary Fig. 10). These results indicate that, in the absence of functional ARU, these proteins are properly targeted to their subcellular compartment. However, we cannot rule out that the extracellular domain of FER is un- or misglycosylated at specific glycosylation sites in *aru*, which may allow the protein to recognize intra- but not interspecific PTs.

The synergid has a specialized secretory region at its micropylar end, the filiform apparatus, which contains a large amount of secreted material and is believed to be the site of PT recognition^6^. It is possible that even subtle differences in ARU protein levels could lead to the misglycosylation of target proteins, including FER, such that a few specific glycosylation sites remain unglycosylated. Given the high secretory activity of the filiform apparatus, such small changes could have a large effect on PT reception. Further work will be necessary to shed more light on the target proteins of *ARU* and to elucidate the role of specific glycosylated surface proteins in PT reception.

## Discussion

**Figure 6:**
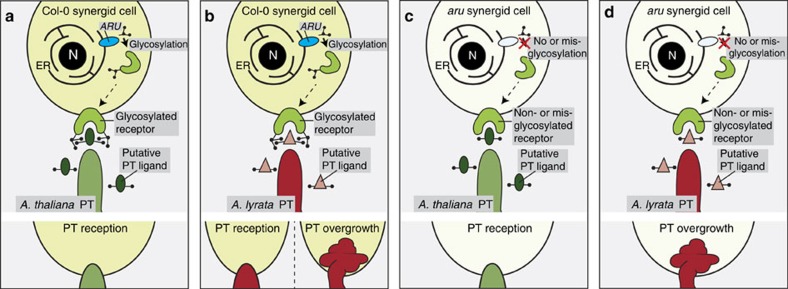
Potential mechanisms of interspecific PT reception and the role of glycosylation. (**a**) In wild-type Col-0 synergid cells, a receptor is glycosylated by ARU and can bind putative signals from the *A. thaliana* PT with its carbohydrate moieties and via protein–protein interactions. (**b**) *A. lyrata* PT signals might not be properly recognized by protein–protein contacts but partially by carbohydrate interactions, thus leading to PT overgrowth in some ovules and normal PT reception in others. (**c**) In *aru* mutants, the synergid receptor is un- or misglycosylated; nevertheless, *A. thaliana* PTs can be efficiently received through protein–protein interactions of the receptor with the PT signals. (**d**) In *aru* mutants pollinated with *A. lyrata* pollen, PT reception is neither possible by carbohydrate, nor by protein–protein interactions. Therefore, almost all ovules show PT overgrowth.

A possible interpretation of our results is that FER, and/or yet unknown synergid (co-)receptors, bind putative ligands from intraspecific PTs both via specific interactions with carbohydrates on the receptor protein and via direct protein–protein interactions, a mechanism similar to the proposed ‘domain-specific model' in mammalian sperm-egg binding^47^ (Fig. 6a). Ligands from interspecific PTs might not be able to sufficiently interact via protein–protein contacts alone but could still be recognized to some extent via the carbohydrate moieties, explaining the partial *A. lyrata* PT reception success in Col-0 ovules (Fig. 6b). It is conceivable that in *aru*, and potentially also in *A. thaliana* accessions with a similar phenotype, changes in the glycosylation status of the receptor could completely abolish the ability to recognize and receive interspecific PTs (Fig. 6d), while ligands from *A. thaliana* PTs are still efficiently recognized via protein–protein interactions, leading to normal PT reception (Fig. 6c).

The crosstalk between gametophytes constitutes a specific form of cell–cell communication. Cellular interactions are often mediated by specific binding of an extracellular ligand to a receptor, triggering downstream signalling cascades in the recipient cell. Most extracellular ligands and receptors are heavily glycosylated^48^, which influences their binding specificities and conformation, such that already the absence of a single glycosylation motif can reduce or abolish a receptor's function and ligand-binding affinity^49^,^50^. Our results suggest that both protein–protein interactions and recognition mediated by carbohydrates may be crucial factors to ensure species-specific PT reception. Thus, divergent evolution of receptor–ligand pairs, as well as of the factors controlling their glycosylation status, could establish new species barriers. Deciphering the molecular basis of speciation in plants might enable us to overcome existing hybridization barriers, which could eventually be of great agronomic importance.

## Methods

### Plant material and growth conditions

The *A. thaliana* accessions were part of the Nordborg collection for GWAS^27^,^40^. Amplified seed stocks were kindly donated by Ortrun Mittelsten Scheid (Gregor Mendel Institute, Vienna). After stratification (2 days at 4 °C), the seeds were allowed to germinate for 6 days on MS plates (22 °C, 16 h light, MS from Carolina Biological Supply). Because some accessions require vernalization, all the seedlings were kept in a vernalization chamber for 5 weeks (4 °C, 16 h light) on MS plates before they were transferred to the soil (ED73, Universalerde).

The accessions were grouped into early- (four incomplete blocks A, B, C and D), mid-, and late flowering plants (three complete blocks A, B, C each) according to the flowering time^27^, and were grown in a greenhouse chamber (22 °C, 16 h light) in an incomplete randomized block design. See Supplementary Table 1 for the assignment of accessions to the blocks.

*A. lyrata*^13^, *A. halleri* (a gift from Marcus Koch, University of Heidelberg), and *A. arenosa* (donated by Matthias Helling, University of Zurich) plants were stratified for 10 days and grown in the same greenhouse chamber. Plants were vernalized to induce flowering (see above).

SALK-lines were obtained from NASC: SALK_067271 (*At1g61790, aru-1*), SALK_137883C (*At1g61780*), SALK_052207C and SALK_026074C (*At1g61795*), SALK_104077 (*At1g61810*). The EMS allele *aru-2* (ref. 34) was a gift from Peter McCourt (University of Toronto). The plants were grown as described before^17^.

### Crosses and aniline blue staining

Flowers were emasculated and the pistils were pollinated 2 days after emasculation (dae). Siliques were collected two days after pollination and fixed for aniline blue staining in 9:1 ethanol:acidic acid. Aniline blue staining was performed as described previously^17^, and the samples were analysed with a Leica DM6000B microscope (Leica Microsystems). For GWAS phenotyping, 9–20 siliques of a minimum of three individuals were analysed for each accession (Exceptions: Zdr-1: seven siliques, Got-7: four siliques from two individuals).

### GWAS analyses

Association mapping was conducted using the mean values of the proportions of ovules with PT overgrowth per silique (OG/S) as phenotypes. An *A. thaliana* 250 K Affymetrix SNP genotyping data set^27^ was downloaded from https://cynin.gmi.oeaw.ac.at/home/resources/atpolydb. GWAS analyses were performed using a compressed mixed linear model, using population parameters previously determined^51^,^52^, and a kinship matrix to account for family relatedness, in the R package GAPIT^53^. The mixed linear models were run with and without principal components as fixed effects to correct for population structure. Multiple testing was controlled using the Bonferroni correction and false-discovery rate^54^. GWAS analyses were also run using a general linear model in the web-based interface TASSEL3.0 (ref. 30) and an accelerated mixed model with Box-Cox transformed phenotypes in GWAPP^55^. MLMM analysis was conducted as previously described^31^.

### Constructs for stable plant transformation

For *pMYB98/pFER::ARU-GFP* the complete coding sequence of *ARU* without the stop-codon was amplified using gene-specific primers with attB-sites for Gateway cloning: 5′- GGGGACAAGTTTGTACAAAAAAGCAGGCTTCATGGCGCTCAAATCAAAACTCGTC -3′ and 5′- GGGGACCACTTTGTACAAGAAAGCTGGGTCACGCCAACTCGATGGCCAATACGGA -3′. We introduced the PCR-fragment into pDONR207, and subsequently into the destination vector (using the *E. coli* strain DH5-alpha F'*I*^*q*^ from New England Biolabs). The destination vector was a modification of the plant Gateway vector pMDC83, which contains the 2x35S-promotor before and *GFP* after the Gateway cassette^56^. For our purpose, we exchanged the original 2x35S-promoter with the promoters of *FER*^13^ and *MYB98* (ref. 35) to express *ARU* specifically in synergid cells. The *MYB98* promoter was amplified from Col-0 with primers 5′- TTTAAGCTTATACACTCATTGTCCTTCG -3′ and 5′- CCCTCTAGATGTTTTGGAAAGGAGAAAAAA -3′, introducing a *Hin*dIII and *Xba*I restriction site, respectively. The *FER* promoter was amplified from the *pFER::FER-GFP* construct^13^ using specific primers 5′- TTTGGTAAGCTTCGATTTAAGCGAG -3′ and 5′- TTTTCTAGACGATCAAGAGCACTTCTCCGGG -3′, which introduce *Hin*dIII and *Xba*I restriction sites as well. The 2x35S-promoter was cut out of pMDC83 (ref. 56) with *Hin*dIII/*Xba*I (New England Biolabs), and the PCR fragments were introduced into the dephosphorylated vector backbone by ligation. pDONR207 carrying the *ARU* coding sequence and the modified destination vector were combined in an LR reaction. The resulting vectors, *pFER::ARU-GFP* and *pMYB98::ARU-GFP* were transformed into *Agrobacterium tumefaciens* strain CV1310, and homozygous *aru-1* plants were transformed by the floral dip method^57^. The complementation assays were conducted in the T2 and T3 generations with plants homozygous for the complementation construct and the *aru-1* mutation. For experiments with *pMYB98::ARU-GFP* in accessions with high OG/S, the construct was transformed into Fei-0, Kz-1, and Kz-9, and OG/S was assessed in heterozygous plants (T1 generation).

For the constructs with *ARU* under the control of its endogenous promoter, the *ARU* fragment (including 1,492 bp of upstream, *ARU* CDS, and 865 bp of downstream sequence) was amplified from Col-0 and Ga-0 genomic DNA using primers 5′- TTTTACTAGTAGGCAATTCCATCAGTTGTT -3′ and 5′- TTTTGGTACCGTTACTTCACTTTCTCGAGT -3′, introducing a *Spe*I and a *Kpn*I restriction site, respectively. The fragments were cloned into pMDC99 (ref. 56) using restriction-ligation and transformed into *aru* mutants (Col-0 background).

*pARU::ARU-GFP* was cloned by amplification of a part of *ARU* coupled to *GFP-tNOS* from *pFER::ARU-GFP* with primers 5′- GCGTTAACGCTTTACCTCA -3′ (including the natural *Hpa*I site in *ARU*) and 5′- TTTGGATCCAGTAACATAGATGACACCGCG -3′ (introducing a *BamH*I site after *tNOS*). This fragment was introduced by ligation into the pMDC99 vector carrying the genomic fragment of *ARU* (Col-0). By cutting this vector with *Hpa*I and *BamH*I, part of the *ARU* coding sequence and the downstream sequence were removed and replaced with the respective fragment of *ARU* coupled to *GFP-tNOS*, resulting in *pARU*(1,492 bp)*::ARU-GFP*, which was transformed into Col-0.

*pLRE::LRE-Citrine* was cloned with overlapping PCR fragments that were assembled using the Gibson cloning Master Mix from New England Biolabs according to the manufacturer's recommendations. The 779-bp long promoter sequence with the predicted signal peptide from *LRE*^16^ was amplified with primers 5′- GTGCTGCAAGGCGATTAAGTCCGTGTGCTCTGTCTGCATT -3′ and 5′- CACAGCTCCACCTCCACCTCCAGGCCGGCCTATGGAACTTGAAGAGGAGAGAGA -3′, introducing an overhang complementary to the vector pMDC99 (ref. 56). *Citrine* was amplified from the transgenic line CS36962 (ordered from Arabidopsis Biological Resource Center, ABRC), using gene-specific primers with overhang primers for the signal peptide of *LRE* and overhang primers for the GPI-anchor of *LRE*: 5′- GGCCGGCCTGGAGGTGGAGGTGGAGCTGTGAGCAAGGGCGAGGAGCT -3′ and 5′- GGCCCCAGCGGCCGCAGCAGCACCAGCAGGATCCTTGTACAGCTCGTCCA -3′. The GPI-anchor of *LRE* was amplified with overhang primers for pMDC99: 5′- TGCTGGTGCTGCTGCGGCCGCTGGGGCCTCGGGTATGTCTTTTTGTTGTC -3′ and 5′- AGCTCCACCGCGGTGGCGGCCGCTCTAGAAGTCTCGCTTCTTCTTTTGT -3′. pMDC99 was amplified with overhang primers for the *LRE* promoter and the GPI-anchor using primers: 5′- ACTTAATCGCCTTGCAGCAC -3′ and 5′- TCTAGAGCGGCCGCCACCGCGG -3′. All the constructs were verified by sequencing. *pFER::FER-GFP* and *pNTA::NTA-GFP* were described previously^13^,^17^.

### ARU-GFP subcellular localization

We used the *pFER::ARU-GFP* construct for microprojectile bombardment of onion epidermal cells and co-localized it with the ER-marker pER-rk (mCherry) obtained from ABRC^58^. Biolistic bombardment of onion epidermis was performed as described^17^.

For visualizing GFP expression in the synergids, flowers were emasculated and pistils were dissected 2 dae to ensure the development of mature, unfertilized embryo sacs. The tissue was mounted on slides in 1 M glycine, pH 9.6. Images were captured on Leica Confocal Microscopes SP2 and SP5 (Leica Microsystems).

### RNA extraction and reverse transcriptase PCR

RNA from pistils (25 pistils, 2 dae), inflorescences, and ovules (extracted from 30 pistils, 2 dae) was extracted using the Trizol reagent (Invitrogen) according to the manufacturer's recommendations. Pistil and inflorescence cDNA was reverse transcribed using Oligo-dT primers and Superscript II reverse transcriptase from Invitrogen. Ovule cDNA was amplified using the Ovation Pico SL WTA system V2 from Nugen.

Reverse transcriptase PCR (RT–PCR) of *ARU* was done using primers 5′- CAATGTGCTTGTTCGAGTG -3′ and 5′- ATCCAGTCTTCCAGTTATCCA -3′. For quantitative RT and digital droplet PCR of *ARU* in *A. thaliana* accessions, the primers 5′- GTTTGTTACCAATGTGCTTGTTCG -3′ and 5′- TCCATATCCAGTCTTCCAGTTATCC -3′ were used and expression levels were normalized against *UBIQUITIN C* (*UBC9*, primers: 5′- ATGCTTGGAGTCCTGCTTGG -3′ and 5′- TGCCATTGAATTGAACCCTCTC -3′). For digital droplet PCR on ovule cDNA, the *UBC9* assay was performed as an EvaGreen assay, whereas *ARU* transcripts were detected using a gene-specific probe (5′-FAM- TACTGCACAAAGGTTG -MGB-3′). The samples were analysed with the QX200 system from Bio-Rad.

### Population genetic analyses and statistical tests

Determination of *ARU* gene structure and UTRs is based on annotations in the ARAMEMNON database^37^. Sequences of *ARU* and 1,000 bp up- and downstream flanking regions were downloaded from http://signal.salk.edu/atg1001/3.0/gebrowser.php. For accessions for which no sequences or only sequences with missing data were available, we amplified the whole region from genomic DNA using primers 5′- TTTGCTATAGGCACATGTGT -3′ and 5′- GACCCGAAATTGTCAAATGA -3′, and sequenced the resulting PCR products of Bay-0, Fab-2, Fab-4, Omo-2-3, Knox-10, Kz-1, LL-0, Lz-0, Mr-0, Mrk-0, Zdr-6. The upstream region was sequenced additionally from Got-7, Pu2-23, and Spr1-6 (primer 5′- TTTGCTATAGGCACATGTGT -3′ and 5′- CGGAGGTTAGGAATTTTGAGA -3′), and the downstream region from Got-7, Pu2-23, Kz-9, Mz-0, Pro-0, Van-0, and Var2-1 (primer 5′- CAATGTGCTTGTTCGAGTG -3′ and 5′- GACCCGAAATTGTCAAATGA -3′). Tajima's D and Fay and Wu's H were calculated separately for the 1,000 bp up- and downstream as well as the coding sequence with the set of 96 accessions^40^ using DnaSP 5.10 (ref. 59). Several accessions that had big indels in the up- and downstream regions were left out from the analysis (Mr-0, Got-7, Pu2-23, and Spr1-6 for the upstream, Var2-1, Nok-3, and Got-7 for the downstream region). *A. lyrata* was used as outgroup. *P* values against the null model were obtained by running 10,000 coalescent simulations and for Tajima's D, the 5% quantile was calculated using previously published estimates for D^40^.

## Additional information

**How to cite this article:** Müller, L. M. *et al*. A subunit of the oligosaccharyltransferase complex is required for interspecific gametophyte recognition in *Arabidopsis*. *Nat. Commun.* 7:10826 doi: 10.1038/ncomms10826 (2016).

## Supplementary Material

Supplementary Information Supplementary Figures 1-10, Supplementary Tables 1-2 and Supplementary References

Supplementary Data 1 Correlation of SNPs (1000 bp downstream region) with OG/S phenotype of accessions
