# Supplementary material for: A subunit of the oligosaccharyltransferase complex is required for interspecific gametophyte recognition in Arabidopsis
Source: Nat Commun. 2016 Mar 11;7:10826. doi: 10.1038/ncomms10826 (PMC4792959; doi:10.1038/ncomms10826)
Supplement: Supplementary Information — Supplementary Figures 1-10, Supplementary Tables 1-2 and Supplementary References [file ncomms10826-s1.pdf]

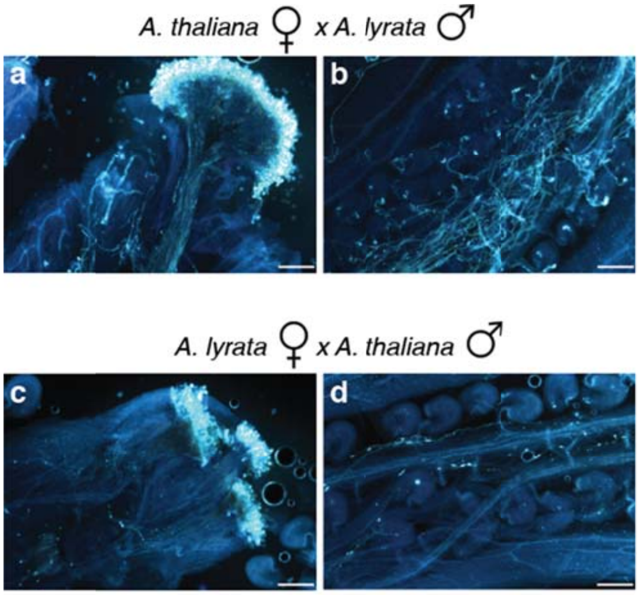

Supplementary Figure 1: Interspecific crosses between *A. thaliana* and *A. lyrata*. (**a**) and (**b**) When *A. thaliana* pistils are pollinated with *A. lyrata* pollen, the PTs germinate (a) and grow through the transmitting tract towards the ovules (b). If they are not recognized correctly, the PTs continue growing inside the female gametophyte (PT overgrowth). (**c**) and (**d**) Pistils of *A. lyrata* pollinated with *A. thaliana* pollen. In this case, the crossing barrier occurs earlier than in the reciprocal cross: most pollen grains do not germinate on the stigma (c), and only a few are visible in the transmitting tract (d). Scale bar 250 µm.

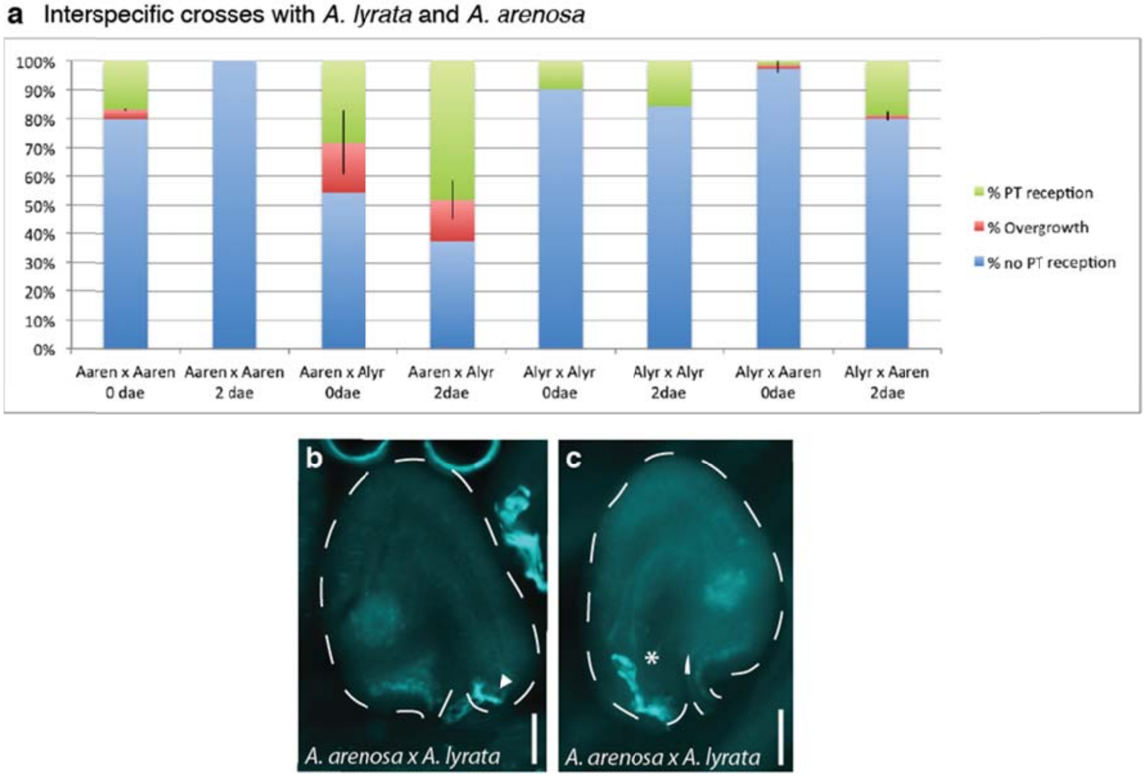

Supplementary Figure 2: Interspecific crosses between *A. lyrata* and *A. arenosa*. Higher proportions of ovules with PT overgrowth are observed in interspecific crosses than in intraspecific crosses (**a**) In all crosses, the majority of the ovules did not attract pollen tubes, possibly due to self-incompatibility mechanisms of the species. (**b**) Example of an *A. arenosa* ovule with normal *A. lyrata* PT reception (arrowhead). (**c**) Example of an *A. arenosa* ovule with *A. lyrata* PT overgrowth (asterisk). Scale bars 50 µm.

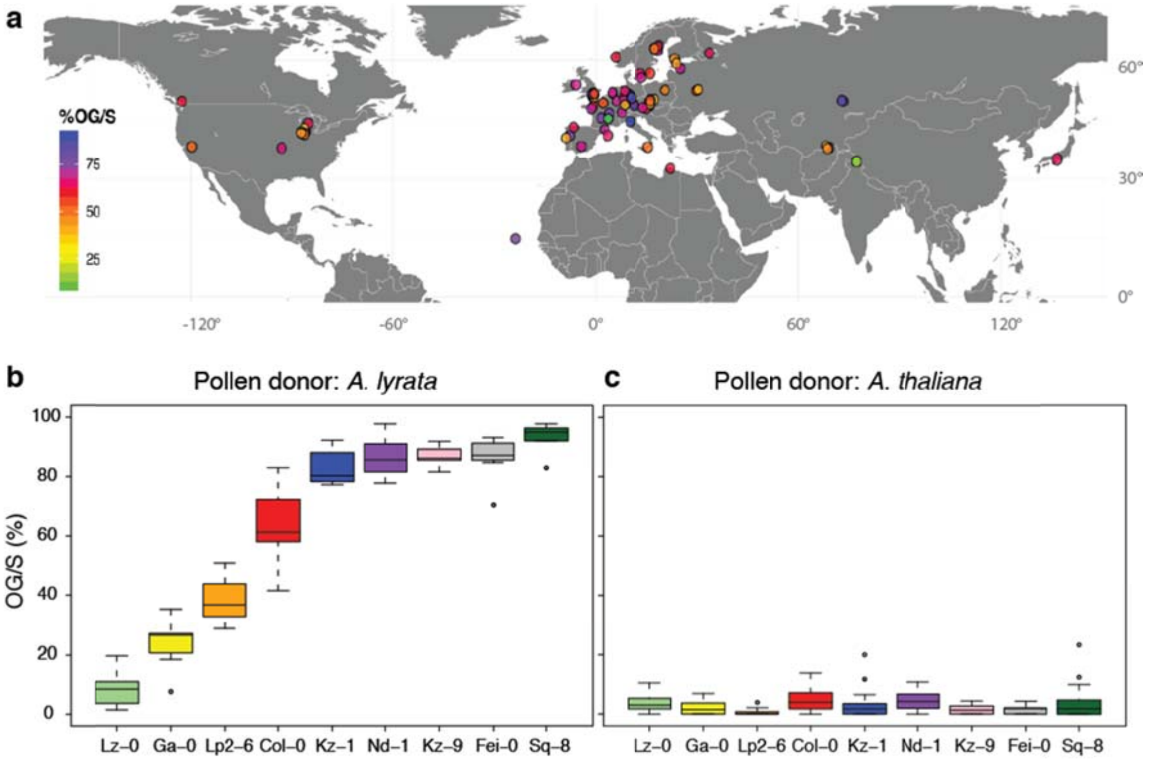

Supplementary Figure 3: Geographical origin of accessions used in this study and their phenotype in inter- and intraspecific crosses. (**a**) Map of the geographical origin of the *A. thaliana* accessions used for phenotyping with their color-coded phenotypes (% OG/S). (**b**) PT overgrowth in a subset of accessions pollinated with *A. lyrata* pollen. (**c**) The same subset pollinated with intraspecific *A. thaliana* pollen. As no difference was observed between *A. thaliana* pollen from low or high OG/S accessions, the data were pooled. All accessions, no matter if they display low or high PT overgrowth phenotypes in interspecific crosses, show normal intraspecific PT reception with only a low level of PT overgrowth.

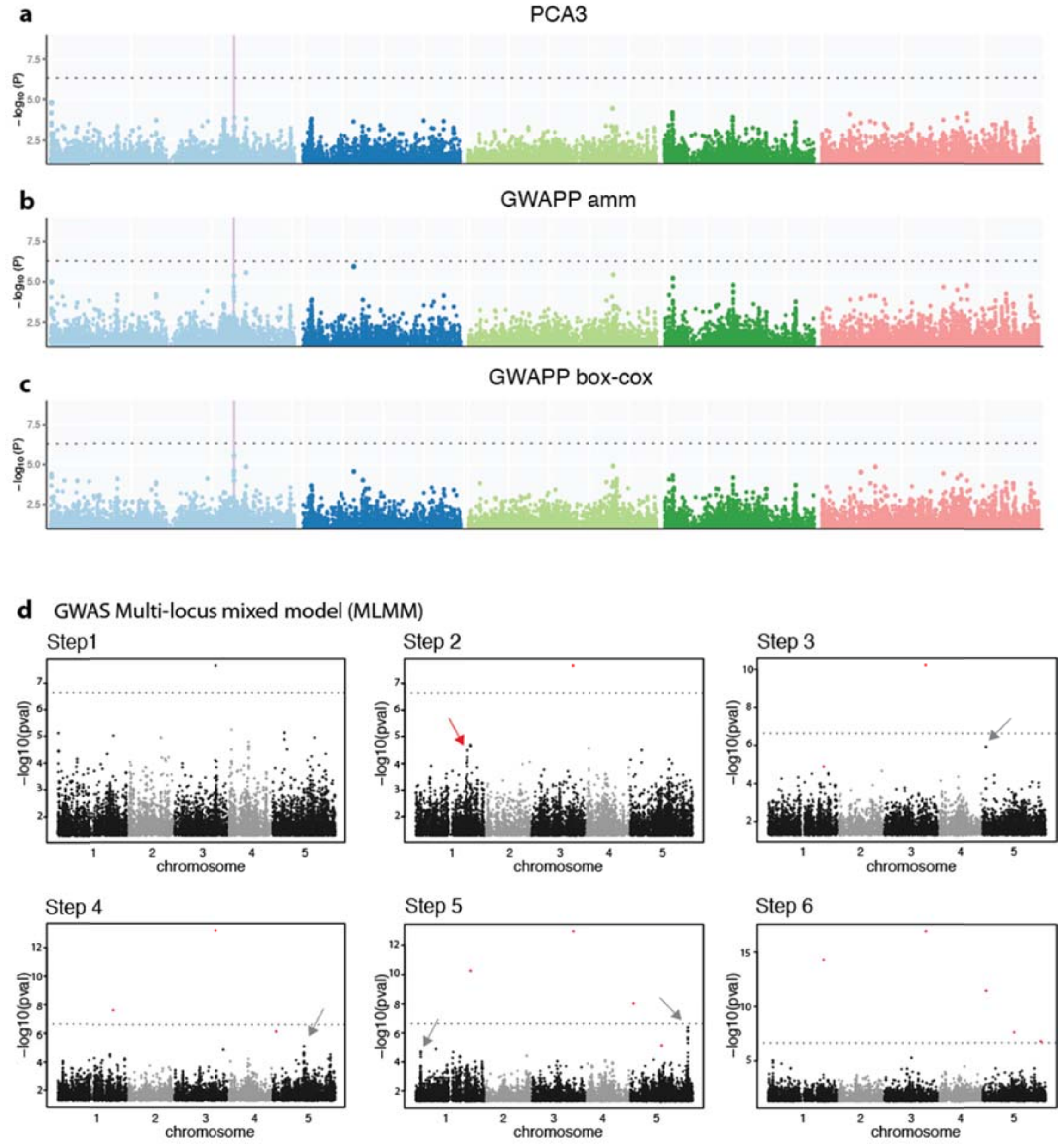

Supplementary Figure 4: Manhattan plots of GWAS analyses with mixed models. (**a**) GWAS calculated with GAPIT (PCA[1]), correcting for population structure and relatedness using principal component analysis (PCA). There is no peak at the *ARU* locus (vertical line). (**b**) and (**c**) Outputs of the GWAS calculated with the web-program GWAPP[2]. With both methods (accelerated mixed model, amm (b) and especially after box_cox normalization (c)), a peak at the *ARU* locus is visible, although it is not significant. (**d**) Output of mixed linear model[3]. Step 1 is calculated with a regular EMMA algorithm. For the following steps, the SNP with highest association is used as a co-factor in the model (red). With each step, new associations are detected (arrows). Red arrow in step 2 points to the *ARU* region. Dotted line: Bonferroni corrected p=0.1 (a-c), and p = 0.05 (d).

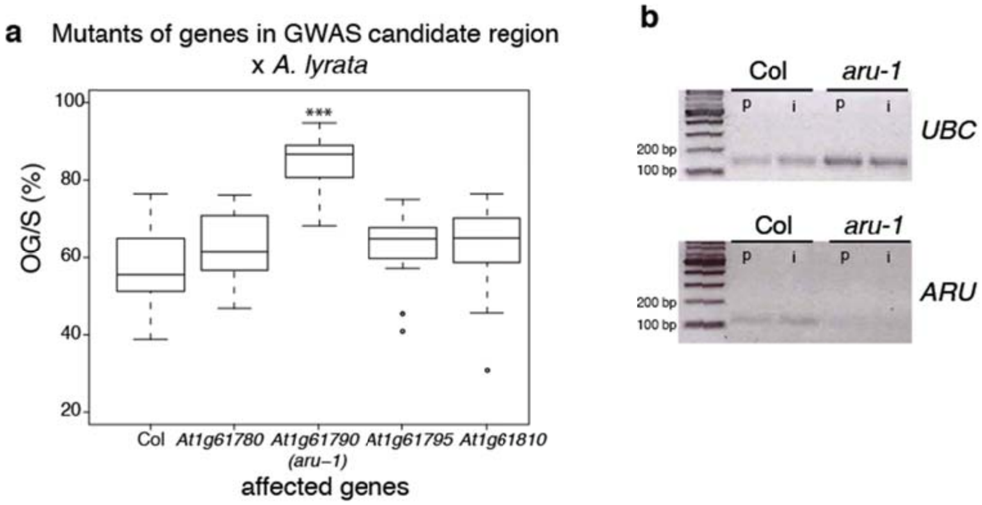

Supplementary Figure 5: Mutant analysis of synergid-expressed genes in the GWAS candidate region on chromosome 1. (**a**) PT overgrowth of candidate mutants in interspecific crosses with *A. lyrata*. Only *aru-1*, a T-DNA insertion in *At1g61790*, shows a phenotype different from wild-type Col-0 (n=19 for Col-0, n=23 for *At1g61780*, n=24 for *aru-1*, n=9 for *At1g61795*, n=60 for *At1g61870*). (**b**) Semi-quantitative RT-PCR showing that *ARU* mRNA is absent in *aru-1*. The housekeeping gene *UBC* was used as control. *** Student's t-test p<0.001.

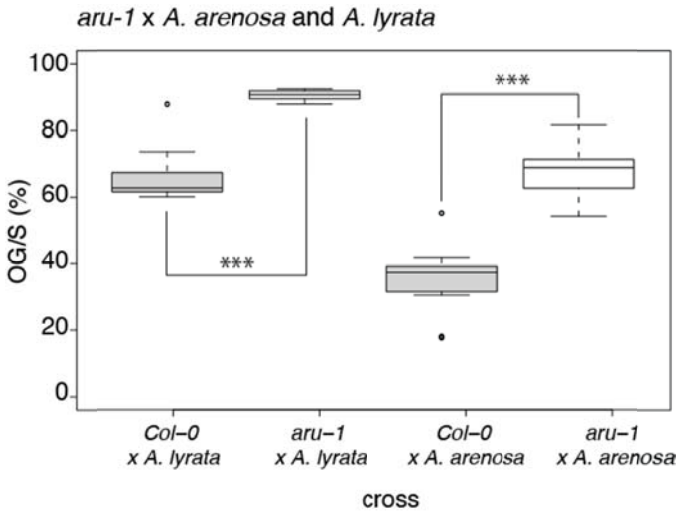

Supplementary Figure 6: *aru* mutants in crosses with *A. lyrata* and *A. arenosa*. *aru-1* mutants show increased OG/S levels compared to Col-0 in crosses with both *A. lyrata* and *A. arenosa* pollen (n=10 for Col-0 x *A. lyrata*, n=8 for *aru-1* x *A. lyrata*, n=12 for Col-0 x *A. arenosa*, n=8 for *aru-1* x *A. arenosa*). *** $p<0.001$ (Student's t-test).

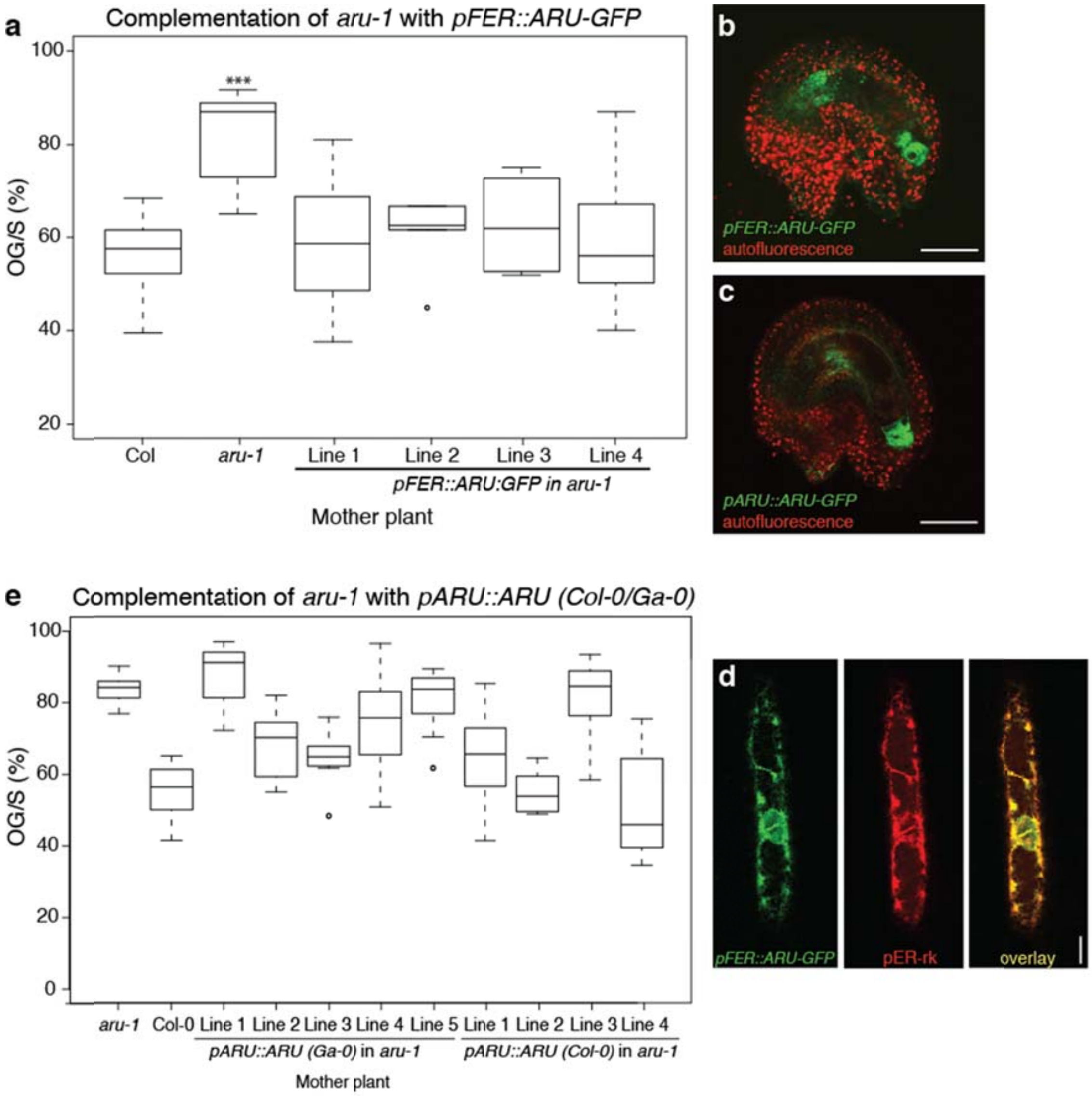

Supplementary Figure 7: *ARU-GFP* expression under the control of the *FERONIA* promoter (*pFER*) and complementation of *aru* with *ARU* alleles from Ga-0 and Col-0. (**a**) PT overgrowth in interspecific crosses with *A. lyrata*. Four lines expressing *ARU-GFP* under the control of the *FER* promoter in the *aru* background that complement the mutant phenotype (n=4-8 siliques per transgenic line). Significance levels in comparison to Col-0 (*** Student's t-test $p<0.001$). (**b**) An ovule expressing *pFER::ARU-GFP*. Expression is strongest in the synergids and ARU-GFP localizes to a perinuclear structure. **c**, An ovule expressing *pARU::ARU-GFP* with strong expression in the synergids. (**d**) Transient expression of FER-GFP (green, from *pFER::ARU-GFP*) and the ER-marker pER-rk (red[4]) in transiently transformed onion epidermal cells. Both markers co-localize, indicating that ARU localizes to the ER. (**e**) Complementation of *aru-1* with the *ARU* genomic region from Ga-0 and Col-0. The Ga-0 allele cannot complement the mutant beyond OG/S levels of Col-0. Scale bars: 50 μm.

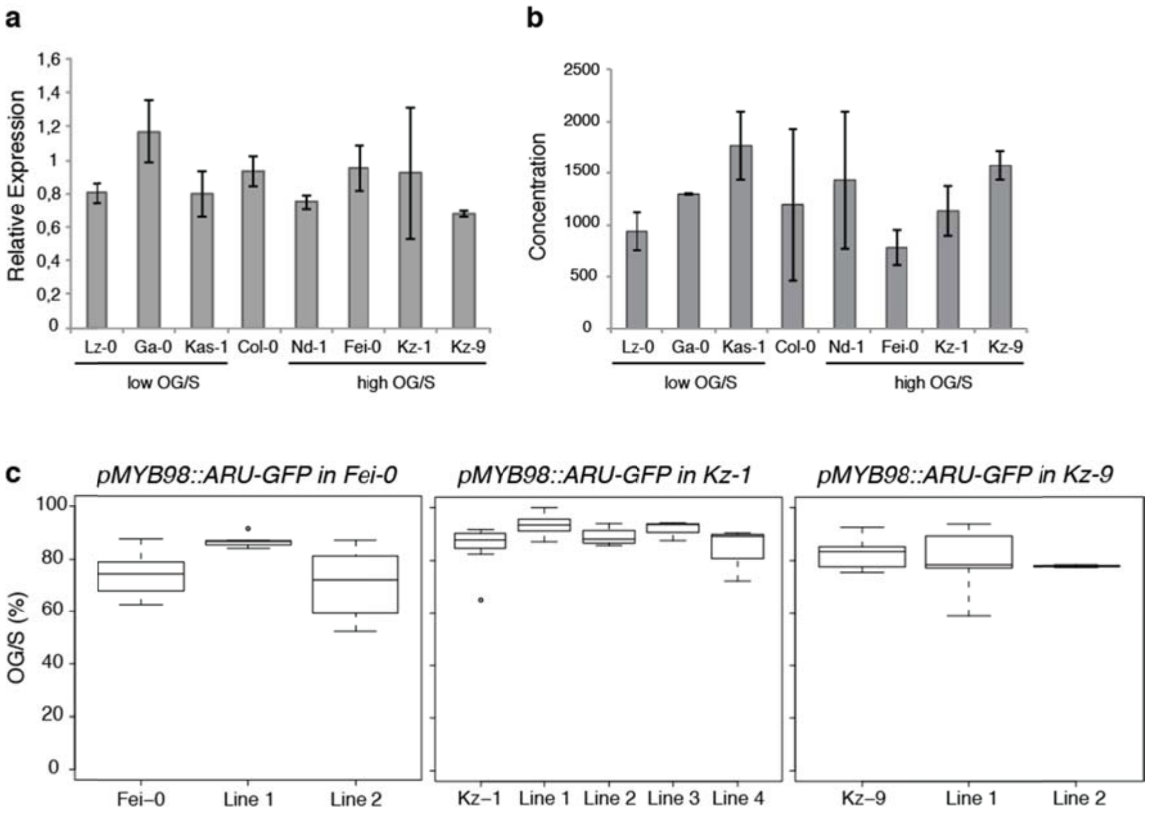

Supplementary Figure 8: *ARU* expression levels in accessions with low and high PT overgrowth in interspecific crosses. (**a**) Relative *ARU* mRNA levels in pistils of accessions with low and high proportions of PT overgrowth in interspecific crosses. All accessions showed similar expression levels of *ARU* and no consistent trend with respect to expression was found. (**b**) Relative *ARU* concentrations in ovule RNA samples assessed with digital droplet PCR. Error bars visualize variation between the two biological replicates that were assessed for each accession. This variation could be caused during sample preparation and/or RNA amplification. Again, no obvious correlation of *ARU* concentrations to OG/S in the accessions was detected. (**c**) Fei-0, Kz-1, and Kz-9 expressing *pMYB98::ARU-GFP* (heterozygous T1 plants) in interspecific crosses with *A. lyrata* pollen. *ARU* expression in the synergids of these accessions does not influence OG/S (n=3-15 siliques).

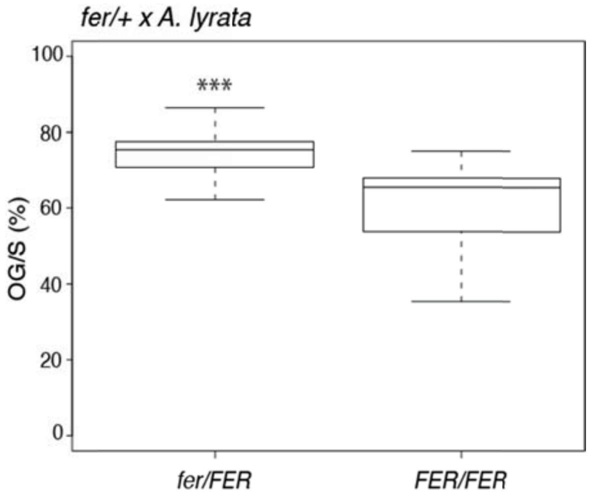

Supplemental Figure 9: *feronia* (*fer*) mutants in interspecific crosses. *fer* heterozygous mutants (*fer/FER*, n=26 siliques) show higher interspecific PT overgrowth than wild-type segregants (*FER/FER*, n=15, *** p<0.001, Student's t-test). Female transmission efficiency of *fer/FER* plants in crosses with *A. thaliana* is 85.5%[5]. Thus, with an OG/S value of 61.2% in wild-type segregants, the expected OG/S in interspecific crosses for *fer/FER* is 73.3%, which is not different from the observed value (74.9%, p>0.1, Chi-square test).

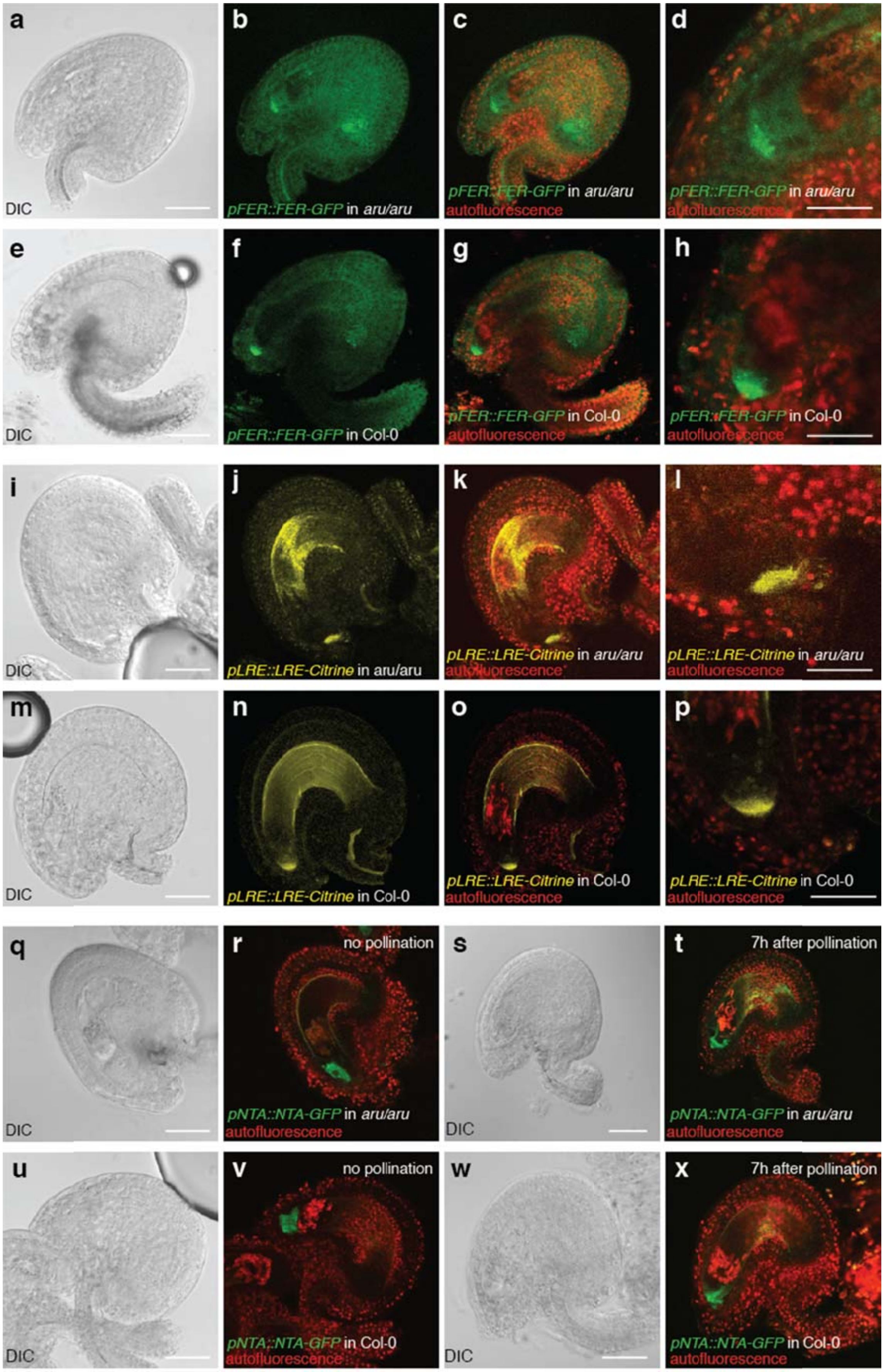

a
DIC
b
pFER::FER-GFP in aru/aru
c
pFER::FER-GFP in aru/aru
autofluorescence
d
pFER::FER-GFP in aru/aru
autofluorescence
e
DIC
f
pFER::FER-GFP in Col-0
g
pFER::FER-GFP in Col-0
autofluorescence
h
pFER::FER-GFP in Col-0
autofluorescence
i
DIC
j
pLRE::LRE-Citrine in aru/aru
k
pLRE::LRE-Citrine in aru/aru
autofluorescence
l
pLRE::LRE-Citrine in aru/aru
autofluorescence
m
DIC
n
pLRE::LRE-Citrine in Col-0
o
pLRE::LRE-Citrine in Col-0
autofluorescence
p
pLRE::LRE-Citrine in Col-0
autofluorescence
q
DIC
r
no pollination
pNTA::NTA-GFP in aru/aru
autofluorescence
s
DIC
t
7h after pollination
pNTA::NTA-GFP in aru/aru
autofluorescence
u
DIC
v
no pollination
pNTA::NTA-GFP in Col-0
autofluorescence
w
DIC
x
7h after pollination
pNTA::NTA-GFP in Col-0
autofluorescence

Supplementary Figure 10: Expression of known factors involved in intraspecific PT reception in *aru* and wild-type ovules. (**a**) to (**d**) *pFER::FER-GFP* in *aru*. (**e**) and (**f**) *pFER::FER-GFP* in Col-0. The strongest FER-GFP expression is detected at the filiform apparatus in synergids both in *aru* and wild-type ovules. (**i**) and (**l**) *pLRE::LRE-Citrine* in *aru*. (**m**) and (**p**) *pLRE::LRE-Citrine* in Col-0. LRE-Citrine is localized exclusively to the filiform apparatus both in *aru* and wild-type embryo sacs. (**q**) and (**r**) *pNTA::NTA-GFP* in *aru* embryo sacs of emasculated pistils. (**s**) and (**t**) *pNTA::NTA-GFP* in *aru* embryo sacs that received a PT (7 hours after pollination). NTA-GFP is found throughout the synergids before fertilization and is re-localized to the micropylar pole upon fertilization. (**u**) and (**v**) *pNTA::NTA-GFP* in Col-0 ovules of emasculated pistils. (**w**) and (**x**) *pNTA::NTA-GFP* in wild-type ovules that received a PT. There is no visible difference to NTA-GFP in *aru*. Scale bars: 50 µm for all images except d, h, l, p: 25 µm.

Supplementary Table 1: *A. thaliana* accessions used in this study. This table shows their origin[6], the blocks they were assigned to, and the mean values of ovules with PT overgrowth per silique (OG/S) that were used for the calculation of the GWAS statistics.

Accession	Region of Origin	Block	Mean values of OG/S (%)
Ag-0	France	Early A, C, D	76.69
An-1	Belgium	Early A, C, D	50.35
Bay-0	Germany	Early B, C, D	57.04
Bil-7	Sweden (North)	Late A, B, C	73.87
Bor-1	Czech Republic	Early A, B, D	46.30
Bor-4	Czech Republic	Early B, C, D	75.56
Br-0	Czech Republic	Early A, C, D	58.51
Bur-0	Ireland	Mid A, B, C	68.08
C24	Portugal	Early B, C, D	39.46
CIBC-17	England	Mid A, B, C	64.46
CIBC-5	England	Early B, C, D	57.62
Col-0	USA (Germany?)	Early A, B, C	65.77
Ct-1	Italy	Early A, B, D	52.68
Cvi-0	Cape Verde Islands	Early A, C, D	75.05
Eden-2	Sweden (North)	Late A, B, C	61.40
Ei-2	Germany	Mid A, B, C	69.39
Est-1	Estland	Early A, B, C	68.34
Fab-2	Sweden (North)	Late A, B, C	73.58
Fab-4	Sweden (North)	Late A, B, C	64.22
Fei-0	Portugal	Early A, B, D	78.14
Ga-0	Germany	Early A, B, C	18.25
Got-22	Germany	Late A, B, C	88.58
Got-7	Germany	Late A, B, C	79.91
Gu-0	Germany	Early A, B, D	60.46
Gy-0	France	Early A, C, D	55.09
HR-10	England	Early B, C, D	75.41
HR-5	England	Early B, C, D	66.29
Kas-1	India	Mid A, B, C	14.81
Kin-0	USA	Early A, C, D	61.82
Knox-10	USA (Indiana)	Mid A, B, C	41.97
Knox-18	USA (Indiana)	Mid A, B, C	59.68
Kondara	Tajikistan	Early A, B, D	56.19
Kz-1	Kazakhstan	Early A, C, D	81.16
Kz-9	Kazakhstan	Early A, B, C	87.30
Ler-1	Poland	Early B, C, D	86.97
Lov-1	Sweden (North)	Late A, B, C	59.19
Lov-5	Sweden (North)	Late A, B, C	46.48
Lp2-2	Czech Republic	Early B, C, D	76.80
Lp2-6	Czech Republic	Early A, B, D	38.89
Lz-0	France	Early A, B, D	10.20

Mr-0	Italy	Late A, B, C	90.93
Mrk-0	Germany	Mid A, B, C	38.10
Mt-0	Libya	Early A, B, D	60.61
Mz-0	Germany	Early B, C, D	70.95
N3	Russia	Mid A, B, C	63.47
Nd-1	Germany	Early A, B, C	88.75
NFA-10	England	Early A, B, C	54.95
NFA-8	England	Early B, C, D	38.30
Nok-3	Netherlands	Mid A, B, C	69.64
Omo2-3	Sweden (South)	Mid A, B, C	58.47
Oy-0	Norway	Mid A, B, C	59.90
Pna-10	USA (Michigan)	Mid A, B, C	33.74
Pna-17	USA (Michigan)	Mid A, B, C	54.57
Pro-0	Spain	Early A, B, D	62.54
Pu2-23	Croatia	Early A, C, D	57.39
Pu2-7	Croatia	Mid A, B, C	65.38
Ra-0	France	Early A, C, D	78.02
Ren-1	France	Mid A, B, C	59.34
Ren-11	France	Early A, B, D	70.88
Rmx-A02	USA (Michigan)	Early A, B, C	44.97
Rmx-A180	USA (Michigan)	Early B, C, D	38.71
RRS-10	USA (Indiana)	Late A, B, C	47.03
RRS-7	USA (Indiana)	Mid A, B, C	45.47
Se-0	Spain	Early A, C, D	68.35
Sha	Tajikistan	Early A, B, D	36.90
Sorbo	Tajikistan	Early A, B, C	46.70
Sq-1	England	Early A, C, D	54.45
Sq-8	England	Early A, B, C	83.45
Tamm-2	Finland	Mid A, B, C	44.85
Tamm-27	Finland	Mid A, B, C	43.77
Ts-1	Spain	Early A, B, D	70.22
Ts-5	Spain	Early B, C, D	63.92
Tsu-1	Japan	Mid A, B, C	62.53
Ull2-3	Sweden (South)	Early A, C, D	64.16
Uod-1	Austria	Early A, B, C	61.70
Uod-7	Austria	Early A, B, C	68.19
Van-0	Canada	Early A, B, D	60.37
Var2-1	Sweden (South)	Late A, B, C	68.16
Wa-1	Poland	Early A, C, D	48.68
Wei-0	Switzerland	Early A, B, C	69.10
Ws-0	Ukraine	Mid A, B, C	56.44
Ws-2	Ukraine	Early A, B, C	37.04
Wt-5	Germany	Mid A, B, C	64.56
Yo-0	USA	Mid A, B, C	51.50
Zdr-1	Czech Republic	Early A, B, D	38.38
Zdr-6	Czech Republic	Early B, C, D	52.47

Supplementary Table 2: Top40 SNPs of the GWAS output (TASSEL GLM[7]) ordered by their p-value

Rank	Marker	Chromosome	Locus_pos	p
1	PERL0219430	1	24325910	3.80E-07
2	PERL0198933	1	22842107	4.85E-07
3	PERL0998055	5	15096264	2.22E-06
4	PERL0198560	1	22816028	2.24E-06
5	PERL0198559	1	22815992	4.21E-06
6	PERL0667236	4	972178	5.61E-06
7	PERL0661920	4	505633	5.63E-06
8	PERL0062096	1	8320546	6.49E-06
9	PERL0198834	1	22836745	7.19E-06
10	PERL0198872	1	22839590	8.05E-06
11	PERL1016861	5	16420039	1.18E-05
12	PERL0166252	1	19503734	1.31E-05
13	PERL0198952	1	22842689	1.34E-05
14	PERL0329435	2	6392760	1.36E-05
15	PERL0198924	1	22841729	1.38E-05
16	PERL0213344	1	23898307	1.55E-05
17	PERL0198538	1	22814316	1.56E-05
18	PERL0907143	5	6679400	1.65E-05
19	PERL0667367	4	978836	1.86E-05
20	PERL0339587	2	7531633	2.19E-05
21	PERL0339588	2	7531688	2.19E-05
22	PERL1101530	5	23812400	2.22E-05
23	PERL1101540	5	23813337	2.22E-05
24	PERL1101550	5	23813943	2.22E-05
25	PERL1101566	5	23815590	2.22E-05
26	PERL1101568	5	23815756	2.22E-05
27	PERL1101569	5	23815796	2.22E-05
28	PERL0291095	2	2220611	2.53E-05
29	PERL0617038	3	18010190	2.58E-05
30	PERL0667057	4	957961	2.72E-05
31	PERL0978994	5	13673830	2.74E-05
32	PERL0277850	2	1030731	3.07E-05
33	PERL1015945	5	16345246	3.14E-05
34	PERL0996614	5	15041429	3.18E-05
35	PERL0219784	1	24354156	3.26E-05
36	PERL0726223	4	6441807	3.31E-05
37	PERL0217577	1	24223030	3.33E-05
38	PERL0233274	1	25610774	3.53E-05
39	PERL0891205	5	4924090	3.57E-05
40	PERL1101497	5	23809336	4.00E-05

Supplementary References

1. Lipka, A. E. *et al.* GAPIT: genome association and prediction integrated tool. *Bioinformatics* **28,** 2397–2399 (2012).
2. Seren, U. *et al.* GWAPP: a web application for genome-wide association mapping in *Arabidopsis*. *Plant Cell* **24,** 4793–4805 (2012).
3. Segura, V. *et al.* An efficient multi-locus mixed-model approach for genome-wide association studies in structured populations. *Nat. Genet.* **44,** 825–830 (2012).
4. Nelson, B. K., Cai, X. & Nebenfuhr, A. A multicolored set of *in vivo* organelle markers for co-localization studies in *Arabidopsis* and other plants. *Plant J* **51,** 1126–1136 (2007).
5. Huck, N., Moore, J. M., Federer, M. & Grossniklaus, U. The *Arabidopsis* mutant *feronia* disrupts the female gametophytic control of pollen tube reception. *Development* **130,** 2149–2159 (2003).
6. Nordborg, M. *et al.* The pattern of polymorphism in *Arabidopsis thaliana*. *PLoS Biol.* **3,** e196 (2005).
7. Bradbury, P. J. *et al.* TASSEL: software for association mapping of complex traits in diverse samples. *Bioinformatics* **23,** 2633–2635 (2007).
